# Polymeric Composite Reinforced with PET Fiber Waste for Application in Civil Construction as a Cladding Element

**DOI:** 10.3390/polym14071293

**Published:** 2022-03-23

**Authors:** Daniel Araujo, Joyce Azevedo, Pollyana Cardoso, Benjamin Lazarus, Matheus Morreira, Lorrane Silva, Josiane Barbosa

**Affiliations:** 1 Department of Materials, University Center SENAI CIMATEC, Salvador 41650-010, BA, Brazil; dvanna.eng@gmail.com (D.A.); pollyanam@fieb.org.br (P.C.); 2 Institute of Science, Technology and Innovation, Federal University of Bahia, Camaçari 42809-721, BA, Brazil; joyce.azevedo@ufba.br; 3 Materials Science and Engineering Program, University of Califórnia San Diego, San Diego, CA 92093, USA; bslazaru@eng.ucsd.edu; 4 Center for Science and Technology in Energy and Sustainability, Federal University of Recôncavo da Bahia, Feira de Santana 44042-280, BA, Brazil; mfalcao1994@gmail.com (M.M.); lorranecarneiro.26@gmail.com (L.S.)

**Keywords:** polymer, composite, PET fiber, industrial waste, cladding, construction

## Abstract

The construction industry contributes enormously to the high levels of carbon dioxide on the planet. For this reason, the sector has been investing in the development of new products that reduce the environmental impact. This study developed a fibrous polymeric composite using industrial residues of polyethylene terephthalate (PET) fibers for application in civil construction as a cladding element. The thermal and morphological characterization of the fiber was performed using Thermogravimetry (TG) and Scanning Electron Microscopy (SEM). Composites with 1, 3, and 5% PET fibers were obtained. Mechanical, morphological properties, chemical resistance, the effect of ultraviolet radiation and water absorption of the composites were evaluated. The results were compared to parameters established by the Brazilian standard NBR 15.575-3. Fibers had a smooth surface but with small surface defects, diameter between 20 and 30 µm and thermal stability up to 325.44 °C. The addition of 5% PET fibers resulted in an increase of more than 300% in the impact resistance of the composites, but with a reduction in the flexural strength. The mechanical and chemical resistance results met the parameters established by the standard used in the study. The degradation chamber test indicated that PET fibers suffered more from exposure to ultraviolet radiation than the polymeric matrix.

## 1. Introduction

The increased levels of carbon dioxide (CO_2_) in the atmosphere are alarming due to its direct influence on the global warming process [1]. A study measuring the levels of global CO_2_ emissions from 1928 to 2017 found that carbon dioxide emissions come primarily from three sources: fossil fuels, deforestation, and carbonate decomposition [2]. The construction industry contributes enormously to the final category. Cement is the most consumed product in the sector, and with an annual production of 4 billion cubic meters in 2019, it is currently the largest source of CO_2_ emissions by carbonate decomposition on the planet. Production of cement has increased by more than 30 times in the last 65 years, largely in China. Since just 1990, its production has increased by more than 11 times, exceeding the global average. It is estimated that by 2060 the number of buildings on the planet will double; meeting this global demand will be the equivalent of rebuilding New York every 30 days [3,4].

Bilal et al. [5] emphasize the importance of developing sustainable products aimed at civil construction to reduce the environmental impact caused by the sector. Lin et al. [6] suggest that the creation of composites consisting of industrial waste can be an excellent alternative for developing ecologically suitable products that contribute to reducing the amount of waste released into the environment.

Composites are defined as a class of material consisting of two or more phases physically and chemically distinct, intentionally arranged or distributed, and which have characteristics that are different from its components in isolation. Three items determine the characteristics of a composite: the reinforcement, the matrix, and the interface between them. These materials are classified based on the type of matrix used (polymer, metal, or ceramic) and reinforcements used (particles, short fibers, continuous fibers, or laminar). Polymer matrix composites generally use carbon, glass, aramid, and polyester fibers as reinforcement. In many non-structural applications, natural fibers are used, such as coconut, jute, sisal, and pineapple fibers [7,8,9].

In this context, the present work aims to develop a fibrous polymeric composite from öindustrial residues of polyethylene terephthalate (PET) fibers for application in civil construction as a cladding element.

Although there is no specific standard for the use of polymeric slabs as a coating in the construction sector, the authors used the Brazilian standard NBR 15575-3 for ceramic slabs in the study as a parameter for the data obtained [10]. As the work is a preliminary study, the authors decided to use the standard of a widely used product.

## 2. Materials and Methods

### 2.1. Materials

**Figure 1:**
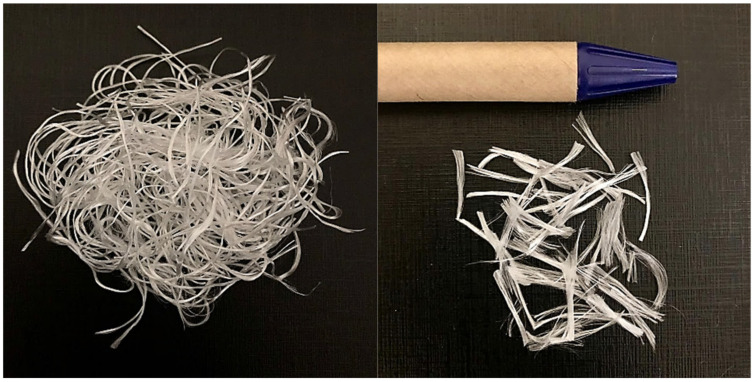
PET fiber used as reinforcement.

For the polymer matrix, an unsaturated polyester resin, AROPOLTM L 50500, was used. This is an accelerated, thixotropic, and low viscosity resin, used for the manufacture of composite parts by open molding as well as Hand Lay Up and Spray Up processes and manufactured by Ashland in EUA. As a curing agent, 1.00 g of BRASNOX^®^ DM-50, manufactured by Nouryon Brazil, catalyst was used for each 100 g of polyester resin, as recommended by the manufacturer. The PET fiber used in the study was provided by the Bahia unit of the multinational Kordsa Global located in the Industrial Pole of Camaçari-Bahia-Brazil. These fibers are a waste by product generated during the manufacturing process of fabrics obtained from high tenacity PET yarns. According to Kordsa, these fabrics can reduce the volume of the rubber compound in tires used in automobiles by up to 30% [11]. The fibers used in the study were cut manually to an average length of 1.1 cm (±0.3 cm), as shown in Figure 1.

### 2.2. Composites Speciments

**Figure 2:**
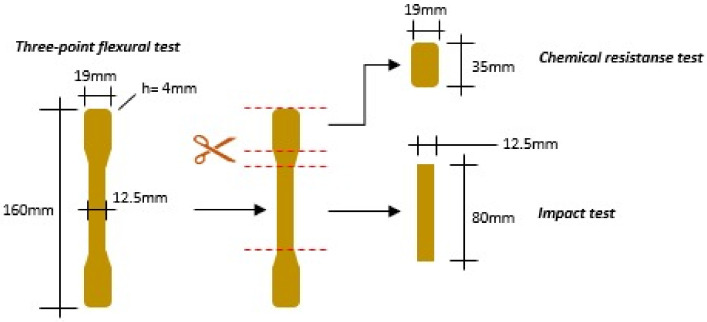
Process for obtaining the samples.

The silicone mold used to prepare the specimens was based on Pereira et al. [12] who used reference samples with the dimensions required by the standard ISO I78 [13]. Composites were mixed manually to have fiber weight percentages of 1, 3, and 5% and randomly oriented fibers. Specimens containing pure resin were also made for comparison during mechanical characterization. Figure 2 depicts how specimens were fabricated for each test beginning with samples that were taken directly out of the silicone mold.

### 2.3. PET Fiber Morphological Characterization

In order to study the surface of the fibers used in the study and identify a possible relationship with the results obtained in three mechanical tests, a JEOL Scanning Electron Microscope (SEM) model JSM-6510 LV (JEOL Ltd., Tokyo, Japan) was used. For the SEM analysis, carbon deposition was performed on the surface of the samples using the Desk V and Carbon Yarn Accessor equipment (Denton Vacuum, Moorestown, NJ, USA).

### 2.4. Composite Characterization

#### 2.4.1. Mechanical Characterization of Composites

The three-point bending test was performed in accordance with ISO 178 [13] in a universal testing machine brand EMIC, model DL 2000, with a 50 kN load cell. A deflection rate of 2 mm/min was used with a distance between the supports of 50 mm. The results correspond to the average of 05 samples tested, with the results being processed in the Software Tesc.

To determine the impact resistance, an Instron machine, model CEAST 9050, with a 2.7 J hammer and IZOD configuration without a notch, was used following the standard ISO 180 [14]. Five samples were used for each fiber weight percentages and were cut to 80 mm in length, 12.5 mm in width and a height of 4 mm. 

#### 2.4.2. Morphological Analysis of Fractured Composites

Morphological analysis of the composite fracture surface was performed using SEM to evaluate the polymer/fiber adhesion.

The fracture surface of the specimens subjected to impact were used for morphological analysis. The samples underwent a process of carbon deposition on the surface, so that it was possible to perform the analysis in SEM. For that, a Jeol brand equipment and model JSM-6510 LV was used.

#### 2.4.3. Chemical Resistance of Composites

**Figure 3:**
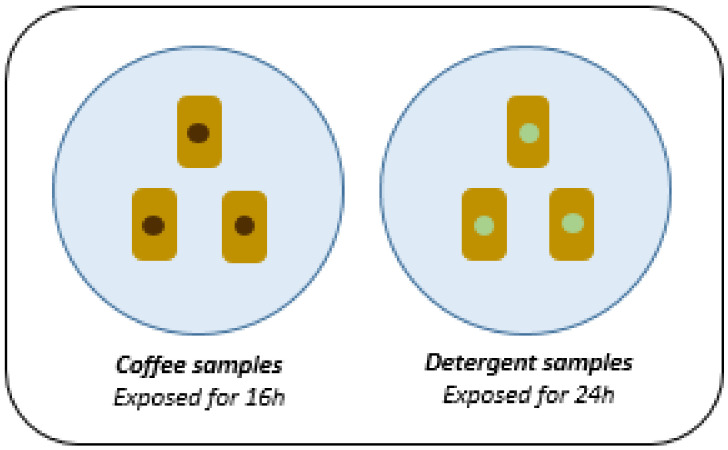
Chemical resistance test.

The chemical resistance test of the composite was based on NBR 15575-3 [10], which recommends the simulation of household cleaning products and chemical agents usually used in the building, followed by a visual assessment of the damage to the coating surface. Six samples were selected with the concentration of fibers that obtained the best results in the impact and bending tests. Half of the samples were exposed to coffee with a concentration of 120 g/L for 16 h and the other containing Ypê brand neutral detergent (Composition: Anionic Surfactants, Sequestrant, Preservatives, Thickener, Dye, Fragrance and Water. Active Ingredient: Linear Sodium Alkyl Benzene Sulfonate) for 24 h, as shown in Figure 3.

To verify the effect of ultraviolet (UV) radiation a degradation chamber was used. 2 samples containing only polyester resin and 2 samples with the formulation that obtained the best mechanical results were selected. The samples were subjected to an 8-h radiation cycle at a temperature of 70 °C for 30 days. The test was performed with the BASS UUV/2009 equipment, following the ASTM G154-12 [15]. After the exposure period, the samples were evaluated using infrared spectroscopy (FTIR) to verify chemical changes. For FTIR analysis, Nicolet iS 10 equipment was used according to international standard ASTM E1252 [16] with reading between 400 and 4000 cm^−1^.

## 3. Results and Discussion

### 3.1. Fiber Morphological Characterization

**Figure 4:**
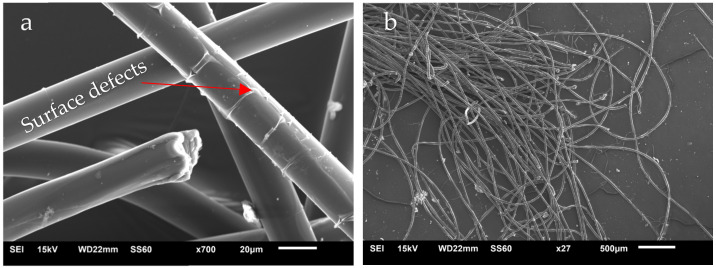
PET Fiber SEM with magnification of: (**a**) 700×; (**b**) 27×.

**Figure 5:**
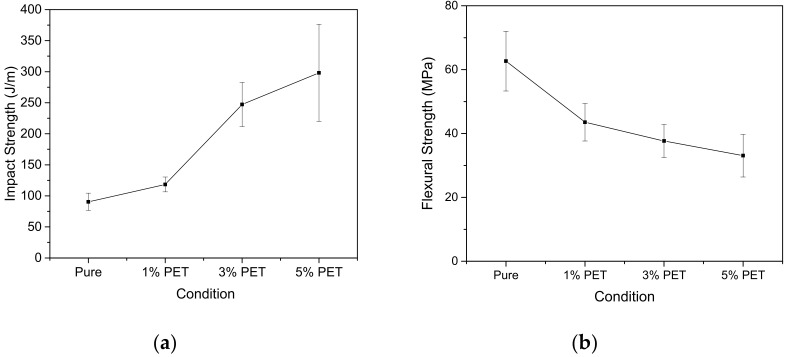
(**a**) Impact resistance; (**b**) Flexural Strength.

Figure 4 shows SEM images of the PET fiber. The fibers had a smooth surface, but with small surface defects. These surface defects can arise during the process of obtaining the fibers or during the drying process to remove moisture from the material [17]. In Figure 5, it is observed that the fibers used in this research have a circular cross section, with a diameter between 20 and 30 µm.

In general, several adhesion mechanisms may be present simultaneously at the interface of a composite depending on the characteristics of the constituents, which may be mechanical interlocking, electrostatic adhesion, interdiffusion and chemical bonding [18]. The presence of defects in the PET fiber can facilitate the penetration and adhesion of the matrix and thus provide better anchoring between the phases [19].

### 3.2. Mechanical Characterization of Composites

The impact and bending strength of the composites are shown in Figure 5. There was a significant increase in the impact strength of composites with the addition of PET fibers. The addition of 5% fibers by weight resulted in an increase of more than 300% and the results suggest that the fibers acted as impact fracture toughness enhancers. Similar results were obtained by Asgari and Masoomi [20]. The authors saw an increase in the impact strength of composites with the increase in the PET fiber content. The difficulty in the molding process of the samples was a limiting factor for the increase in fiber contents. The addition made the homogeneous distribution of fibers complex and favored the appearance of voids in the samples. Possibly, these manufacturing flaws increased the standard deviation of the material strength.

The increase in the fiber volume fraction led to a reduction in bending strength (Figure 5b). The 5% PET fiber composites showed a 47% reduction compared to the sample composed of pure polymer. This result can be explained by the appearance of voids during the sample molding process. Zarate et al. [21] report that voids act as stress concentration points in the composite, leading to material rupture. Impact resistance is a complex combination of strength, stiffness and toughness. In general, more tenacious systems are also more resistant to impact, but this property strongly depends on the ability of the increase in the strength of the system to overcome the losses observed in its deformation. The presence of voids, resulting from the failure in the process of obtaining the composites, influenced the load absorption capacity, which reduced the properties under bending. In addition, composites made up of randomly arranged fibers can be considered isotropic and effectively increase impact resistance through the proper distribution of energy throughout the structure [7,22].

Vardai et al. [23] also studied composites containing PET fibers and an increase in impact strength was observed, indicating that both the initiation and propagation of cracks are impaired by the presence of the synthetic fiber.

The cladding elements in civil construction are mostly connected to a rigid base. Therefore, the claddings are not substantially required to resist bending, but their exposure to the environment makes them susceptible to impacts. The NBR 15575-3 [10] establishes that the cladding must resist effects of 30 J. The composites that were 5% PET Sfibers by weight exhibited values 10× higher than those set by the standard. Regarding flexural strength, the same standard recommends that the cladding has a minimum flexural strength of 10 MPa. Even with the observed drop in the flexural strength of the composite with the increase in fiber contents, the studied formulations satisfactorily met the standard’s requirements.

### 3.3. Morphological Analysis of Fractured Composites

The fracture surfaces of the polymer matrix of the composites were evaluated with scanning electron microscopy. This approach makes it possible to assess the interface between the constituents of the composites. The interface is responsible for transferring mechanical demand between the matrix and the fiber. Therefore, it can be influenced by some factors such as chemical affinity, mechanical anchoring, and manufacturing processes [8].

**Figure 6:**
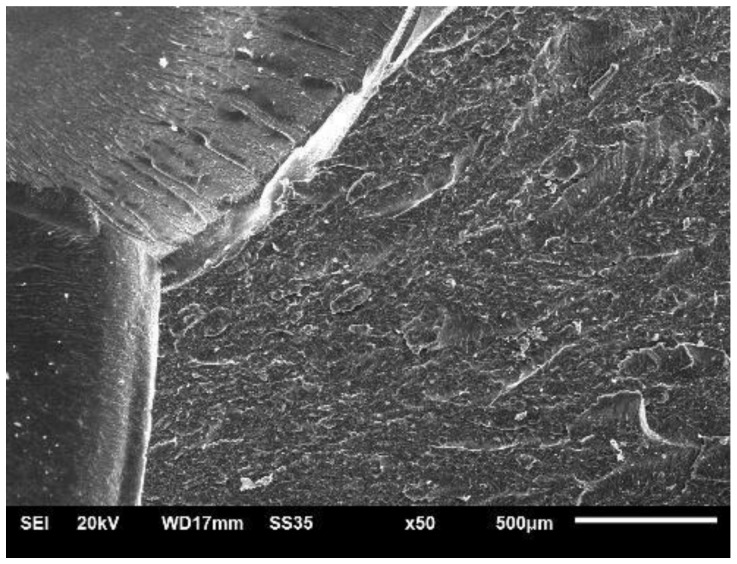
Topography of pure polyester resin fracture.

Figure 6 shows the fracture surface microscopy of a pure polyester resin specimen. It is possible to observe characteristics of a fracture without appreciable and relatively flat plastic deformation.

**Figure 7:**
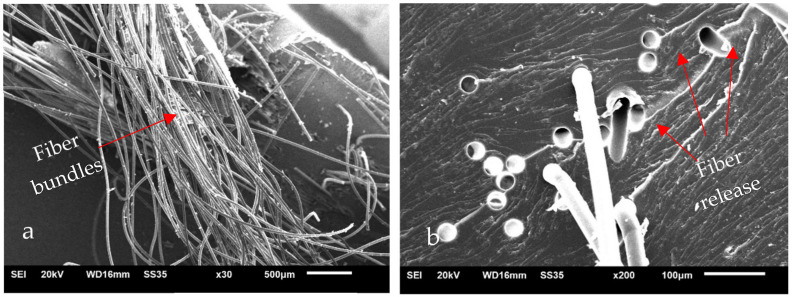
Scanning Electron Microscopy of PET 5% composite fractured surfaces. (**a**) PET fiber bundle; (**b**) PET fiber release.

Figure 7a,b shows the fracture surface of the composite with 5% PET fiber. Figure 7a shows fiber bundles, which suggests poor fiber distribution. This configuration favors the formation of voids and stress concentrations that are susceptible to fracture. Figure 7b shows holes with a diameter and shape similar to that of the fibers, indicating detachment likely occurred due to low anchorage with the matrix.

The SEM images indicated that the irregular fiber distribution during the shaping of the samples possibly contributed to the formation of voids and fiber bundles, resulting in a decrease in the bond strength between the reinforcement and the composite matrix and an increase in the standard deviation of the mechanical tests. New forming methodologies for void mitigation, better fiber distribution and use of compatibilizers must be studied in order to achieve better results.

### 3.4. Chemical Resistance of Composites

**Figure 8:**
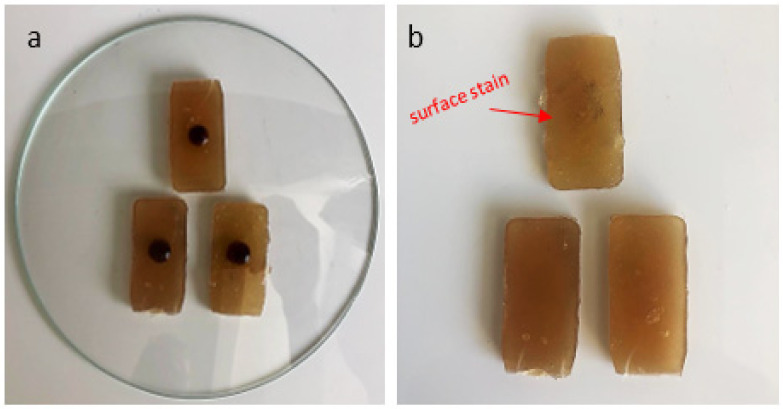
(**a**) A PET 5% samples exposed to coffee at a concentration of 120 g/L for 16 h; (**b**) test result.

**Figure 9:**
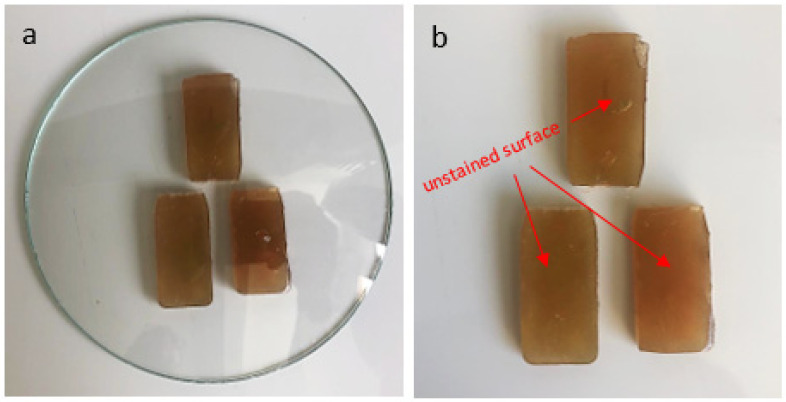
(**a**) A PET 5% samples exposed to detergent neutral detergent for 24 h; (**b**) test result.

Figure 8 and Figure 9 show the results of the chemical resistance test of samples exposed to coffee at a concentration of 120 g/L for 16 h and to neutral detergent for 24 h.

Table 1 presents the visual classification of NBR 15575-3 [10] for qualitative analysis of the results presented in the chemical resistance test. Following the standard criteria, the coffee samples showed damage levels 3 and 4, while the models exposed to detergent did not show visible changes, reaching level 4. The standard does not establish minimum levels to be achieved by the material. It only establishes criteria for better qualification of the results.

### 3.5. Behavior of Composites to Ultraviolet Exposure

This work seeks the development of a composite with a polyester matrix and PET fiber for an application in civil construction, knowing that exposure to ultraviolet radiation (UV) is common in these applications and a possible degradation process, samples of the pure polymer and the composite were subjected to accelerated aging in a chamber.

The degradation of polymeric materials is associated with the oxidation process, resulting in discoloration of the samples, reduced flexibility, cracks and brittle surfaces [24,25]. In polyester composites, when there is exposure to an energy source (solar or thermal) the energy absorbed by the polymer matrix results in the formation of free radicals that can produce a series of decomposition products, including aldehydes and ketones, which can influence mechanical properties [26].

**Figure 10:**
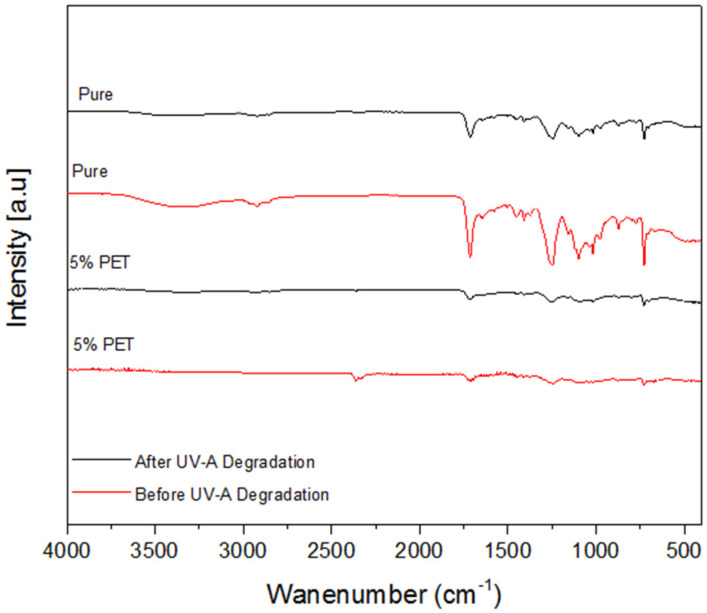
Infrared Spectroscopy of samples containing pure resin and 5% PET fibers before and after degradation chamber.

Figure 10 shows the absorption spectrum of samples containing pure resin exposed to UV radiation and shows the results of samples containing 5% PET fibers.

The spectrum obtained for the unaged pure polyester resin shows a strong spectral band at 729 cm^−1^ and a weak band at 1101 cm^−1^ which can be attributed to the vibration of the –CH group of positions 1 and 3 in the benzene ring and vibration of –C=CH. A spectral band is observed at 1101 cm^−1^ that confirms the presence of the ester bond –C–O–C and a strong peak at 1249 cm^−1^ that can be attributed to the –C=C– group. A spectral band at 1448 cm^−1^ that can be attributed to the vibration of –C–H. The presence of –C=O and symmetrical elongation of –CH were confirmed by the presence of bands at 1708 cm^−1^ and 2923 cm^−1^, respectively. At 3341 cm^−1^ there is a spectral band referring to the vibrations of the −OH group [26].

It is possible to observe a similarity in the graphs (before and after UV degradation) of samples containing polyester resin. The formation of new peaks that would indicate degradation was not observed. However, a reduction in the intensity of the spectral bands of the samples is observed after degradation.

According to Santos et al. [26], in general, the PET spectra are characterized by the broadband between 2200 and 3400 cm^−1^, corresponding to the –OH group linked to the carbonyl. Spectral band before UV degradation is observed at 2357 cm^−1^ in samples containing 5% PET Fibers. However, after the degradation chamber the samples with 5% of PET fibers suffered a decrease in the bandwidth between 2200 and 3400 cm^−1^, and begin to resemble the graph of samples containing only polyester resin.

## 4. Conclusions

A fibrous polymer composite was developed using PET fiber residues for application in civil construction as a coating element using the NBR 15557-3 standard as a reference. The morphology of the fibers indicated a material with a smooth surface and some superficial defects. The addition of 5% PET fibers resulted in an increase of more than 300% in the impact strength of the composites, but with a reduction in the flexural strength. The results of mechanical and chemical resistance met the parameters established by the standard used in the study. The degradation chamber test indicated that the composites with PET fibers suffered more from the effects of exposure to ultraviolet radiation than the polymer matrix. Samples of PET fibers, from industrial waste, have the potential to be used as reinforcement in composites with a polyester matrix. However, it is suggested the development of studies addressing other types of processing and obtaining the specimens to mitigate the formation of voids in the samples, in addition to allowing the incorporation of higher fiber contents.

## Figures and Tables

**Table 1:** Visual assessment of cladding surface damage. NBR 15575-3, 2013 [10].

Damage Levels	Description
4	No visible changes.
3	Slight to moderate in brightness and/or color, visible at any angle of observation.
2	Severe change in brightness and/or color, but no surface attack.
1	Surface attack in the form of cracks, fissures or bubbles.

## Data Availability

Not applicable.
